# The applicability of measures of socioeconomic position to different ethnic groups within the UK

**DOI:** 10.1186/1475-9276-8-4

**Published:** 2009-02-27

**Authors:** Margaret Kelaher, Sheila Paul, Helen Lambert, Waqar Ahmad, George Davey Smith

**Affiliations:** 1 Centre for Health Policy, Programs and Economics, School of Population Health, University of Melbourne, 207 Bouverie St, Carlton Vic 3010, Australia; 2 UCL Centre for International Health and Development, Institute of Child Health, 30 Guilford St, London, WC1N 1EH, UK; 3 Department of Social Medicine, University of Bristol, Canynge Hall, Whiteladies Road, Bristol, BS8 2PR, UK; 4 Social Policy Research Centre, Middlesex University, Trent Park, Bramley Road, London, N14 4YZ, UK

## Abstract

**Background:**

In this paper we seek to tease out differences in socioeconomic position between ethnic groups. There are 3 main reasons why conventional socioeconomic indicators and asset based measures may not be equally applicable to all ethnic groups:

1) Differences in response rate to conventional socioeconomic indicators

2) Cultural and social differences in economic priorities/opportunities

3) Differences in housing quality, assets and debt within socioeconomic strata

**Methods:**

The sample consisted of White (n = 227), African-Caribbean (n = 213) and Indian and Pakistani (n = 233) adults aged between 18 and 59 years living in Leeds as measured in a stratified population survey. Measures included income, education, employment, car ownership, home ownership, housing quality, household assets, investments, debt, perceived ability to obtain various sums and perceived level of financial support given and received.

**Results:**

Response rates to education and income questions were similar for the different ethnic groups. Overall response rates for income were much lower than those for education and biased towards wealthier people. There were differences between ethnic groups in economic priorities/opportunities particularly in relation to car ownership, home ownership, investment and debt. Differences in living conditions, household assets and debt between ethnic groups were dependent on differences in education; however differences in car ownership, home ownership, ability to obtain £10 000, and loaning money to family/friends and income from employment/self employment persisted after adjustment for education.

**Conclusion:**

In the UK, education appears to be an effective variable for measuring variation in SEP across ethnic groups but the ability to account for SEP differences may be improved by the addition of car and home ownership, ability to obtain £10 000, loaning money to family/friends and income from employment/self employment. Further research is required to establish the degree to which results of this study are generalisable.

## Background

Research on ethnic differences in health and other social indicators has been criticised for failing to adequately take into account socio-economic differences between groups. [1-8] More recent work has taken socio-economic position (SEP) into account, [9-13] assuming that once SEP is considered any group differences left are due to factors more intimately linked to ethnicity. This is only true if all necessary factors are taken into account and measures do not allow for substantial residual confounding.[3,13,14] Research using the Fourth National Survey of ethnic minorities in the UK suggests that the use of some conventional measures of socioeconomic position is unlikely to address such confounding. For example, a study of variation within occupational social class groups suggested that ethnic minorities had lower incomes than their white counterparts.[13,14] Similarly research in the US has found that calculating SEP on the basis of income understates the true magnitude of ethnic group differences in economic resources, because there are large ethnic group gaps in wealth at every income level. [15]

The issue of how SEP and ethnicity are related is further vexed by major shifts in indicators of SEP, with much greater emphasis on how traditional indicators like income and education translate operationally into assets, living conditions and resources that may directly impact on health. This is reflected in asset based surveys such as Breadline Britain [16] and the use of items like car ownership, home ownership and central heating from the 2001 Census.[17] However this has not simplified the process of teasing out the contribution of socio-economic circumstances to differentials in health. There is evidence that there are ethnic group differences in income, education and asset based measures. For example in the Fourth National Survey of ethnic minorities while, the Indian and Pakistani group were more likely to own homes, the quality of housing was lower than that of their white counterparts.[13]

In this paper we seek to improve understanding of the appropriate use of measures of socioeconomic position for studying health differentials between ethnic groups in the UK. There are three main reasons why conventional socioeconomic indicators and asset-based measures may not be equally applicable to all ethnic groups:

1) Differences in response rate to conventional socioeconomic indicators;

2) Cultural and social differences in socioeconomic position including work, investment and access to funds;

3) Differences in housing quality, assets and debt within socioeconomic strata.

The paper addresses these issues in three sections. The first section examines ethnic group differences in response rates to questions about education and income. The second section examines ethnic group differences in housing quality, assets and debt. The third section explores whether ethnic group differences in living conditions, assets and debt vary by level of education.

## Methods

### Sample

The survey of white (n = 227), Indian and Pakistani (n = 233) and African Caribbean (n = 213) adults aged 18–59 years living in Leeds, UK was part of a larger study which aimed to examine the relationship between ethnicity, health and SEP carried out as part of the Economic and Social Research Council Health Variations Program. Details of the study sample have been reported previously.[18] Electoral wards were divided into three groups by Townsend score, which combines measures of unemployment, car ownership, home ownership and household crowding. Seven high, medium and low deprivation wards were selected. General practice lists were seen to provide the most appropriate, reliable and up to date sampling frame. In addition, such lists contained the information needed to stratify the sample by age-band and gender. A total of 30 practices were selected; of these 20 agreed to give us access to their practice lists for sampling purposes. The 10 practices that declined to participate were not markedly different than those that did. The practice response rate did not vary between wards.

There is no statutory requirement for primary care to collect data on ethnicity. In the absence of ethnic monitoring, different approaches needed to be taken to identify people to be included in the white sample and the Indian and Pakistani sample, compared to the African-Caribbean sample. In the current study, names were used to allocate individuals to the White and the Indian and Pakistani groups from which random samples were drawn. Patients with South Asian names were identified with the aid of a software package (*Nam Pehchan *sensitivity 88–96% and Positive predictive validity of 59–67% against names from Yorkshire), [19] with additional manual confirmation by team members familiar with South Asian names. In the current study, patients were allocated to the African-Caribbean sample based on recall by practice staff.

Following difficulties in reaching the required numbers, additional interviews were undertaken using quotas to ensure that the sample had an appropriate level of representation of each group. This involved targeting potential interviewees living around a number of identified sampling points through random walks as well as snowball sampling in the electoral wards. Response rates for the pre-selected sample were 27.1% and 41.3% for the quota sample. The overall response rate was 33%. The proportion of people selected by each recruitment method varied between ethnic group (Primary sample: white = 42.5%, Indian Pakistani = 57.1%, African Caribbean = 50.5%). There was no interaction between ethnicity and sample strategy on any socioeconomic variable except home ownership. Among white people the primary sample were slightly more likely than people in the quota sample to own their own homes but the opposite was true for minority ethnic groups.

The ethnicity designation of participants was confirmed using self-identified categorisations that were then mapped into the 2001 census categories. The results are presented here for White, African-Caribbean groups, and, due to small numbers, for Indians and Pakistanis as one group. People of mixed origin were generally assigned to minority rather than white groups. Of the 247 White participants, 241 identified as British, 2 as Irish, 1 as French and 3 as other White. Of the 232 Indian and Pakistani participants, 92 identified themselves as Indian, 129 as Pakistani, 3 as African, 2 as any other Asian background, 1 as Any other mixed background; 1 as British, 1 as British Sikh 1 as East African Asian and 2 as White and Asian. Of the 212 African-Caribbeans, 167 identified themselves as Caribbean, 21 identified themselves as African, 3 as Black British, 7 as British Caribbean and 6 as of other black backgrounds. The remainder identified themselves as being of mixed origin, including 8 as White and Black Caribbean. Each person was only allowed to be in one category. People identifying in two categories where one was a minority were allocated to the minority group. Small sample size meant that there were only sufficient numbers for three groups; White, African Carribbean and Indian and Pakistani. This may limit the generalisability of the results and potentially subgroup differences within ethnic groups.

### Instruments and Procedure

The instrument used in the community survey was developed following preliminary analysis of the qualitative data that had been collected in the previous phase of the research and a detailed review of existing published and unpublished survey instruments used to assess socioeconomic position. The questionnaire included sections on ethnicity, socioeconomic position, social resources, discrimination and health.[18] Demographic and health questions were informed by the Census and Health Survey for England.[17] Household and individual income questions were taken from Breadline Britain.[16] An Urdu version of the questionnaire was available where required.

The survey was conducted by a commercial market research company using face to face interviews to complete each questionnaire. Interviewees and interviewers were matched on the basis of language and gender. Most interviews took place in people's homes.

### Analysis

All analyses were conducted in Intercooled Stata version 10.0. Binomial logistic and linear regressions were used to examine ethnic differences in the response rate to income and education (secondary or below, post-Secondary (non-university) and university) questions by home ownership, car ownership, savings of more than £1000 and can not afford household goods. All SEP measures were coded dichotomously.

Binomial logistic and linear regressions were used to examine ethnic differences in car ownership, home ownership, poor quality housing, worry about losing home, can not afford household goods, investments, no debts, able to get £10 000, owe money to family/friends, lent money to family/friends, welfare and employment/self-employment. All variables were coded dichotomously. People who reported that their house was too damp, uncomfortably cold in winter or too small were classified as having poor housing quality. The measure for can not afford household goods was based on whether the respondent said their household lacked the following goods because they could not afford it; telephone, washing machine, freezer/fridge, dishwasher, mobile phone, cable/satellite television, video recorder, central heating, tumble drier/washer, burglar alarm, compact disc player or home computer. Investments included personal pension, PEP/TESSA/stocks/shares, savings, jewellery, property other than home, community savings scheme and other. Debts included credit card, catalogue, hire purchase, bank loan, family/friend loan, other loan, overdraft and mortgage/rent arrears. The welfare measure included family benefit, income support, job seekers allowance, housing benefit.

Further analysis was conducted to assess the effect of ethnic difference on these SEP measures taking education into account. Rates and means adjusted for age and sex were calculated using adjprop and adjmean procedures. Age was coded into three categories (18–29, 30–44, 45–59 years). Age was further reduced to two categories for some variables, because there were no cases among younger people. Age (and, where appropriate gender) adjusted rates as well as odd ratios are reported, to clarify differences between groups.[5] The white group was used as the reference group for the analysis of ethnic group differences to enable comparison with other studies. Thus p values represent differences between the minority ethnic groups and the white group. Reported differences were significant at the 0.05 level.

## Results

### Response by interviewees to education and income questions

The response rate to the question about highest level of education was 97%. Although there appeared to be a slightly lower response rate for the Indian and Pakistani group this difference was associated with a greater number of first generation immigrants among the Indian and Pakistani group.

Across all ethnic groups studied only 42.2% of people responded to questions about personal income and 22.1% responded to questions regarding household income. There were no substantial differences in response rates between ethnic groups: White 46.1% (95%CI 40.0–52.3%), Indian and Pakistani 37.7% (95%CI 31.8–44.1%) and African Caribbean 37.8% (95%CI 31.8–44.1%).

People who responded to the personal income question were more likely than people who did not respond to own a car (OR = 2.5, 95%CI 1.5–4.2), a home (OR = 2.4, 95%CI 1.4–4.1) and to be able to obtain £1000 from their savings (OR = 2.4, 95%CI 1.3–3.8) regardless of ethnicity. However there were no differences in being able to afford household goods (OR = 0.8, 95%CI 0.5–1.4).

Overall the results suggest that missing responses to income questions are not independent of SEP in any ethnicity. Accordingly education may be a better than income at measuring socioeconomic status across ethnic groups.

### Ethnic Differences in socio-economic position

The Indian and Pakistani group was more likely than the White group to own one car (see table 1), while the African Caribbean group was less likely than the White group to own 1 or more cars. The Indian and Pakistani group was more likely than the white group to own their home (see table 1) but the opposite was true for the African Caribbean group. There were no group differences in rates of people living in poor quality housing (see table 1). Minority ethnic groups were also more likely than the white group to worry about losing their home (table 1). Indians and Pakistanis were less likely than people in the white to report that they could not afford household goods. There were no other group differences.

**Table 1:** Education, assets, debt and sources of income by ethnicity

	**Ethnicity Age sex adjusted (95%CI)**		
	White	Indian & Pakistani	African Caribbean
	N = 247	N = 233	N = 212

**Education**			
Secondary or below	58.1 (57.2–59.1)	71.9 (71.1–72.7)	70.5 (69.8–71.4)
Above Secondary	14.3 (13.9–14.6)	17.4 (16.9–17.9)	16.9 (16.5–17.4)
University	27.6 (26.9–28.2)	10.7 (10.4–11.0)	12.5 (12.1–12.8)
**Living conditions and household assets**			
%Car ownership	54.9 (48.7–61.1)	61.2 (54.8–67.3)	32.4 (26.4–39.0)
%Home ownership	57.2 (50.7–63.4)	71.8 (65.6–77.3)	44.1 (37.3–51.0)
%Poor quality housing	53.4 (47.1–59.5)	63.1 (56.7–69.0)	60.5 (53.7–66.8)
%Worry about losing home	9.1 (6.1–13.4)	18.2 (13.7–23.8)	16.7 (12.1–22.2)
% Can not afford household goods	35.6 (30.1–41.9)	27.0 (20.9–33.0)	40.6 (34.3–47.0)
**Debt and Equity**			
% Investments	77.1 (71.1–81.9)	69.0 (62.7–74.7)	66.0 (59.3–62.1)
% No Debts	44.3 (38.2–50.7)	63.9 (57.5–69.9)	53.6 (46.9–60.3)
%Able to get £10 000	61.7 (55.4–67.6)	52.2 (45.8–58.7)	37.9 (31.6–44.7)
% lent money to family/friends	41.7 (35.7–48.0)	41.2 (35.1–47.7)	54.6 (47.8–61.1)
**Sources of Income**			
%Welfare	18.0 (13.7–23.3)	19.6 (15.0–25.2)	32.6 (26.7–39.2)
%Employment/self-employment	62.6 (56.4–68.5)	51.4 (45.1–58.0)	41.5 (35.0–48.4)

People in both minority ethnic groups were significantly less likely than the white group to report that they had investments (see table 1). Both minority ethnic groups were less likely to than the white group report that they had debt (see table 1). This was particularly true of Indians and Pakistanis. African Caribbeans were more likely than the white group to say that they had lent money to family and friends. There were no differences in lending money to family and friends among Indians and Pakistanis compares to the white group. The Indian and Pakistani and African Caribbean groups were less likely than the white group to report that they could get £10 000 from a bank and more likely to report that they could not get £10 000 (see table 1). Respondents from minority ethnic groups were less likely than the white respondents to report earnings from employment/self employment, and were more likely to report receiving welfare (see table 1).

### The relationship between ethnicity and education and other measures of socio-economic position

Table 2 shows that people with a university education were more likely than people educated at secondary school level or below to own a car (OR = 3.3, 95%CI 1.7–6.2). The Indian and Pakistani group were more likely than the white group to own a car (OR = 2.0, 95%CI 1.2–3.2) and the African Caribbean group were less likely to own a car than the white group (OR = 0.5, 95%CI 0.3–0.9). There was a significant interaction between education and car ownership for the Indian and Pakistani group in comparison to the white group (OR = 0.2, 95%CI 0.1–0.8, see table 2). The university educated Indian and Pakistani group was less likely than the Indian and Pakistani group educated to secondary or below to own a car. The opposite was true for the white group.

**Table 2:** Measures of socioeconomic position by ethnicity and education

**Ethnicity**	**Level of Education**		
	**<=secondary**	**>secondary**	**University**

**% car ownership (95%CI)**			

White	45.7 (37.4–54.2)	63.5 (46.1–78.1)	73.5 (61.4–82.9)
Indian & Pakistani	62.7 (54.4–70.3)	64.7 (47.4–78.8)	57.1 (35.8–76.0)
African Caribbean	29.0 (22.1–37.1)	29.0 (16.2–46.4)	63.1 (42.9–79.6)

**% home ownership (95%CI)**			

White	48.4 (39.8–57.1)	70.4 (52.4–83.6)	77.2 (65.3–85.9)
Indian & Pakistani	70.9 (62.7–78.0)	68.9 (51.3–81.3)	82.6 (60.9–93.5)
African Caribbean	39.0 (31.1–47.5)	46.8 (30.2–64.1)	72.7 (52.1–86.8)

**% poor quality housing (95%CI)**			

White	49.5 (41.1–57.9)	54.5 (37.7–70.4)	54.8 (42.6–66.6)
Indian & Pakistani	61.2 (52.9–68.9)	73.5 (56.4–85.6)	47.6 (27.8–68.1)
African Caribbean	61.0 (52.7–68.7)	65.2 (47.9–79.1)	48.0 (29.7–67.0)

**% worried about losing home (95%CI)**			

White	11.5 (7.1–18.1)	5.9 (1.5–20.8)	7.9 (3.3–17.6)
Indian & Pakistani	20.1 (14.3–27.4)	8.6 (2.7–23.5)	28.3 (13.2–50.4)
African Caribbean	17.9 (12.4–25.1)	18.6 (8.6–35.7)	11.5 (3.7–30.4)

**% investments (95%CI)**			

White	70.2 (61.8–77.4)	88.0 (72.0–95.5)	86.4 (75.7–92.8)
Indian & Pakistani	67.6 (59.4–74.9)	79.7 (62.9–90.8)	86.1 (64.4–95.8)
African Caribbean	58.9 (50.5–66.8)	77.4 (60.6–88.4)	87.9 (68.5–96.1)

**% no debts (95%CI)**			

White	55.1 (46.6–63.3)	21.1 (10.4–38.2)	36.4 (25.5–48.8)
Indian & Pakistani	66.1 (57.8–73.3)	62.1 (45.0–76.6)	62.2 (40.6–80.0)
African Caribbean	55.7 (46.9–63.3)	51.2 (34.7–67.4)	40.7 (23.5–60.4)

**% able to get £10 000 (95%CI)**			

White	50.9 (42.4–59.4)	63.5 (45.9–78.1)	83.3 (72.1–90.6)
Indian & Pakistani	47.7 (39.6–56.0)	67.6 (50.2–81.1)	81.2 (59.0–92.8)
African Caribbean	33.6 (26.2–41.8)	44.1 (28.4–61.3)	54.3 (34.8–72.5)

**% lent money to family/friends (95%CI)**			

White	43.3 (35.2–51.9)	54.6 (37.6–70.4)	37.1 (26.2–49.5)
Indian & Pakistani	43.1 (35.2–51.4)	43.8 (28.3–60.6)	37.7 (20.0–59.3)
African Caribbean	56.2 (47.9–64.1)	55.1 (47.9–64.1)	51.4 (32.4–69.9)

**% Can not afford household goods (95%CI)**			

White	36.9 (28.5–44.7)	27.3 (14.9–44.7)	36.0 (25.2–48.3)
Indian & Pakistani	25.3 (18.8–33.1)	32.4 (19.0–49.6)	10.0 (24.0–31.2)
African Caribbean	44.0 (36.0–52.3)	44.2 (28.7–61.0)	20.2 (8.7–40.3)

**% earnings from employment/self-employment**			

White	49.0 (40.6–57.5)	88.0 (72.0–95.5)	81.7 (70.4–89.4)
Indian & Pakistani	56.9 (48.5–64.8)	59.0 (41.9–74.1)	71.8 (49.6–86.9)
African Caribbean	36.8 (29.2–45.1)	44.8 (29.1–61.7)	71.8 (51.4–86.0)

Home ownership increased with education (>Secondary: OR = 2.6, 1.1–6.0, University: OR = 3.6, 95%CI 1.8–7.1). The Indian and Pakistani group were more likely than the white group to own their homes at all levels of education (OR = 2.5, 95%CI 1.5–4.1). There was no interaction between ethnicity and education and home ownership.

Poor quality housing was not related to education (>Secondary: OR = 1.1, 95%CI 0.5–2.4, University: OR = 1.2, 95%CI 0.6–2.2, p = 0.7). The Indian and Pakistani group were more likely than the white group to report having poor quality housing (OR = 2.0, 1.2–3.3). There were no clear differences between the African Caribbean group and the white group. There were no differences in worry about losing home either due to education or ethnicity.

The proportion of people with investments increased with level of education (>Secondary: OR = 3.1, 95%CI 1.0–9.5, University OR = 2.7, 95%CI 1.2–6.0). People educated to above secondary school level were less likely to have no debts than people with lower levels of education (>Secondary: OR = 0.2, 95%CI 0.1–0.5, University: OR = 0.5, 95%CI 0.3–0.9).

University educated people were more likely than people educated to secondary level or below to be able to obtain £10 000 (OR = 4.8, 95%CI 2.3–10.1). There were no other significant differences due to education or ethnicity nor were there any significant interactions.

The ability to afford household goods was relatively consistent across levels of education (>Secondary: OR = 0.7, 95%CI 0.3–1.5, University OR = 1.0, 95%CI 0.5–1.8). There were no differences in being able to afford household goods due to ethnicity.

People educated above secondary level were more likely that people educated below secondary level to receive income from employment/self employment (>Secondary: OR = 7.6, 95%CI 2.5–23.0, University: OR = 4.6, 95%CI 2.3–9.5). African Caribbeans were less likely than the white group overall to receive income from employment/self employment (OR = 0.6, 95%CI -0.4–0.9). There were no differences between Indians and Pakistanis and the white group. There was an interaction between ethnicity and education with the apparent benefits of being educated above secondary level in terms of receiving income from employment/self employment being far smaller for the African Caribbeans (OR = 0.2, 95%CI 0.05–0.7) and the Indian and Pakistani (OR = 0.1, 95%CI 0.04–0.5) groups than the white group.

## Discussion

Overall the results suggest that response rates to conventional indicators were relatively consistent across ethnic groups. Response rates to questions about education were high across ethnic groups. On the other hand there was a tendency in all ethnic groups for people who had fewer assets to be less likely to respond to income questions. This effect was more marked in the white group. This suggests that income might be a biased indicator in all ethnic groups unless considerably better response rates can be achieved than in this study.

The extent to which education reflects socioeconomic well-being may depend on the proportion of immigrants in different ethnic communities or at least the extent to which immigrants' qualifications are obtained in the UK. Accordingly the results of this study in regard to education may not be generalisable to samples with high proportions of immigrants or people whose qualifications were obtained outside the UK. There was also some evidence of ethnic variability in perceptions of the usefulness of educational qualifications in gaining employment. It should also be noted that there are many other factors such as household composition that might modify the SEP effects observed.

There were clear differences between ethnic groups in home ownership, a finding consistent with extant literature.[18,20] The results suggested that the Indian and Pakistani group were more likely than the white group to own their own homes. There were few differences in being able to afford household goods, an area where differentials have narrowed over the years.[20,21] The white group generally reported higher levels of debt than people in both other ethnic groups. This suggests that ownership assets and goods alone should not be used as a measure of wealth. However assets may still be useful measures where they are directly related to outcomes being studied. For example, educational outcomes may relate to computer ownership.

Previous research has noted the importance of informal economy and 'support in kind' to working class, including South Asian families. [22] Informal financial exchange is seen to be a key component of family relationships within the Pakistani community. [23] Counter-intuitively, we found no differences between Pakistani and Indian and white in lending money to family and friends. However, in spite of fear of family breakdown, expressed by African Caribbean communities themselves, [24] our findings demonstrate the continued importance of family members in providing financial support.

Differences in socioeconomic indicators by ethnicity were examined within education categories. In general controlling for education did account for most of the variation in SEP between ethnic groups. Differences in assets, debt and investments did not persist when education was taken into account nor were there any significant interactions. Some differences in car and home ownership, ability to obtain £10 000, loaning money to family/friends and income from employment/self employment did persist. This suggests that these variables, in addition to education, might provide an effective basis for capturing socioeconomic position in different ethnic groups. However it should be noted that the sample size in the current study was relatively small limiting our ability to estimate the degree of residual confounding.

## Conclusion

Overall the results support the contention that the relationship between ethnicity and socioeconomic position is complex.[13,15] Response rates to education and incomes were similar for the different ethic groups. Response rates for income were much lower than those for education. Income responses were also biased with wealthier people being more likely to respond. There did appear to be cultural and social differences in economic priorities/opportunities, particularly in relation to car ownership, home ownership, investment and debt. There were limited differences in ownership of household assets. There were also differences in working conditions. Differences in living conditions, household assets and debt between ethnic groups became small once education was taken into account. Education appears to be an effective variable for measuring variation in SEP across ethnic groups but the ability to account for SEP differences may be improved by the addition of car and home ownership and ability to obtain £10 000, loaning money to family/friends and income from employment/self employment. Further research is required to establish the degree to which results of this study are generalisable.

## Competing interests

The authors declare that they have no competing interests.

## Authors' contributions

MK conducted the analysis and drafted the manuscript. SP participated in the design and management of the study. HL, WA, GDS assisted in the design of the study and helped draft the manuscript.
